# Deficit of Cross‐Frequency Integration in Mild Cognitive Impairment and Alzheimer's Disease: A Multilayer Network Approach

**DOI:** 10.1002/jmri.27453

**Published:** 2020-11-26

**Authors:** Xiaoyue Wang, Xiaohong Cui, Congli Ding, Dandan Li, Chen Cheng, Bin Wang, Jie Xiang

**Affiliations:** ^1^ College of Information and Computer Taiyuan University of Technology Taiyuan China

**Keywords:** multilayer, fMRI, frequency, Alzheimer's disease, mild cognitive impairment, hubs

## Abstract

**Background:**

Studies at specific frequencies have shown abnormalities in brain functional networks among mild cognitive impairment (MCI) and Alzheimer's disease (AD) patients. Previous studies have failed to take into account the possibility that optimal cognitive integration requires interactions between different frequency bands.

**Purpose:**

To study whether there is abnormal cross‐frequency integration in patients' brains during disease progression.

**Study Type:**

Retrospective.

**Population:**

Forty‐six normal control (NC), 85 patients with MCI, and 31 patients with AD.

**Field Strength/Sequence:**

3T.

**Assessment:**

Multilayer network models were constructed for NC, MCI, and AD, and multilayer participation coefficient (MPC) was used to study the changes of the interlayer relationship in the course of disease development. In addition, MPC and an overlapping degree were combined to classify nodes in the network, and the role of key nodes in the interlayer interaction was mainly observed. Finally, the correlation between multilayer network measures and cognitive function was investigated.

**Statistical Tests:**

Pearson chi‐squared two‐tailed test, one‐way analysis of variance (ANOVA), nonparametric Spearman correlation coefficient *r*, and the false discovery rate.

**Results:**

The MPC of the network decreased significantly in MCI (*P* < 0.05) and AD (*P* < 0.05). The number of intralayer nodes increased significantly (MCI [*P* < 0.05], AD [*P* < 0.05]) and the number of interlayer nodes decreased significantly. Centrality loss between frequencies of a large number of hub nodes, among which the damaged hub nodes included the left hippocampus, left precuneus, right precuneus, left posterior cingulate gyrus, left precentral gyrus, right precentral gyrus, left medial superior frontal gyrus, and right postcentral gyrus. MPC was significantly associated with memory impairment in patients (AD [Spearman's *r =* 0.526, *P* < 0.05], MCI [Spearman's *r =* 0.229, *P* < 0.05]), and these related regions included damaged hub nodes in patients.

**Data Conclusion:**

In the multilayer networks of patients, there was an obvious deficit in cross‐frequency integration and the hub nodes were preferentially damaged. Moreover, these vulnerable hubs are associated with patients' cognitive scores.

**Level of Evidence:**

1

**Technical Efficacy Stage:**

3

ALZHEIMER'S DISEASE (AD) is a common neurodegenerative disease and has been of great interest to the network neuroscience community because it can cause serious progressive cognitive and functional impairment.^1^ Research has shown that mild cognitive impairment (MCI) is an early stage of AD.^2^ Therefore, exploring the neural relationship between AD and MCI may provide an opportunity for the early diagnosis of AD.

Most functional magnetic resonance imaging (fMRI) studies have demonstrated the gradual deterioration of brain functional networks from MCI development to AD.^3^, ^4^ In these studies, brain signals are usually bandpass‐filtered between 0.01 and 0.1 Hz. However, studies have shown that functional brain networks are frequency‐dependent.^5^, ^6^ Some studies have reported that signals interact or modulate between different bands to support cognitive functions.^7^, ^8^, ^9^ Moreover, a study has shown that the presence of network connections and hub nodes fluctuates in the 0.01–0.1 Hz frequency band.^10^ The importance of each region fluctuates dramatically due to frequency changes, and hub nodes may be very different when functional connections are measured in different frequency bands.^11^ This study shows that the network in a specific frequency range is not a separate entity. The interaction between frequencies should be taken into account in future study.^12^

Therefore, multilayer networks have recently been used to study the complex cognitive activity of the human brain.^13^ In terms of centrality, hubs identified in multilayer networks are different from those identified in monolayer networks, and the use of these brain regions to classify schizophrenia patients and healthy subjects achieved higher accuracy and sensitivity than traditional single‐layer networks.^14^ Dynamic topological changes in the separation and integration of AD brain networks have been explored previously in an individual frequency band.^15^ This research showed that compared with the networks of normal control (NC), those of AD patients have reduced global information processing (reduced interaction between modules) and increased local information processing (increased interaction within modules). It also reveals that a decrease in the number of hubs between modules and an increase in the number of hubs within modules are also observed among nodes, indicating preferential impairment of hub nodes in the patient brain network. These results indicate that AD patient's brain networks tend to feature increased isolation and reduced integration. In addition, hub nodes have indeed been shown to be damaged in AD.^16^ Electrophysiological study has repeatedly shown that cross‐frequency coupling is a mechanism of interaction between different frequency layers.^17^ There is evidence that coupling may occur between different frequency bands in the process of cognition.^18^ However, in multilayer networks it has not been explored whether patients with AD have an abnormal balance of intrafrequency (ie, multilayer networks tend to be isolated) and interfrequency (ie, multilayer networks tend to integrate) information exchange.

The purpose of this study was to explore the trend of information integration ability in patients' brain networks using a multilayer network model, which may help to understand the neural mechanism of different frequency interactions in resting brain networks. In addition, hub nodes in single‐layer networks have been proven to be high‐risk factors in AD.^19^ However, it is not clear whether hub nodes were damaged in multilayer networks. Therefore, this research focused on the core nodes in the network.

## Materials and Methods

### Participants

The resting‐state fMRI data from this study were obtained from the Alzheimer's Disease Neuroimaging Initiative (ADNI) public database. Data collection was conducted according to Good Clinical Practice guidelines, US 21CFR Part 50‐ Protection of Human Subjects, and Part 56‐Institutional Review Boards (IRBs) / Research Ethics Boards (REBs), and pursuant to state and federal regulations. Written informed consent and HIPAA authorizations for the study was obtained from all participants and/or authorized representatives and the study partners before protocol‐specific procedures were carried out. This study included 46 NC subjects (74 ± 6 years, 18 males), 85 patients with MCI (71 ± 8 years, 41 males), and 31 patients with AD (73 ± 7 years, 13 males).

### Data Acquisition

The project used in this study was the ADNI2 dataset in the ADNI database, mainly studying MRI and resting‐state fMRI data of NC, MCI, and AD subjects. Resting‐state fMRI data were obtained from all subjects using a 3T Philips Medical Systems (Best, Netherlands) MR scanner. During the process of data acquisition, the subjects lay supine in the scanner with their eyes closed; the head was stabilized as much as possible to avoid artifacts caused by shaking, and the subjects tried to avoid thinking in any systematic way. The equipment parameters for the acquisition of T_1_‐weighted magnetization prepared rapid gradient echo (MP‐RAGE) sagittal images were as follows: repetition time (TR) = 6.8 msec, echo time (TE) = 3.16 msec, slice thickness = 1.2 mm, flip angle (FA) = 8°, sagittal slices = 170, matrix = 256 × 256, field of view (FOV) = 256 × 256 mm^2^. All resting‐state functional images were collected by an echo‐planar imaging (EPI) sequence. The scanning parameters were as follows: number of slices = 48; TR = 3000 msec; TE = 30 msec; slice thickness = 3.3 mm; FA = 80°, number of timepoints = 140, voxel size = 3 × 3 × 3 mm^3^, matrix = 64 × 59, FOV = 212 × 198.75 mm^2^. The subjects meeting all the above scanning parameters were obtained and the data were checked. Among them, one NC subject was excluded because the timepoint was incomplete, and the other eligible subjects were preprocessed. Table 1 shows the demographic information of the participants.

**TABLE 1:** Subject Characteristics and Cognitive Scores

Group	NC	MCI	AD	*P*‐value
*n*	46	85	31	—
Mean age ± SD	74 ± 6	71 ± 8	73 ± 7	0.126^a^
Gender (F/M)	28/18	44/41	18/13	0.577^b^
Mean MMSE score ± SD	28.9 ± 1.2	26.0 ± 1.7	22.6 ± 2.5	—

F = females; M = males; *n* = number of subjects; SD = standard deviation; NC = normal control; MCI = mild cognitive impairment; AD = Alzheimer's disease, MMSE = Mini‐Mental State Examination. Age and MMSE score are presented as the mean and standard deviation.

^a^ One‐way ANOVA.

^b^ Pearson chi‐squared two‐tailed test.

### Data Preprocessing

The resting‐state fMRI data were processed using the Data Processing Assistant for Resting‐State fMRI (DPARSF) toolbox (http://pub.restfmri.net/), Statistical Parametric Mapping (SPM12) and the Resting‐State fMRI Data Analysis Toolkit (REST 1.8) package were on a MatLab (R2016b, MathWorks, Natick, MA) platform.^20^, ^21^ Data from the first 10 timepoints were removed. The data from the remaining 130 timepoints were preprocessed using DPARSF software as follows: 1) slice timing correction: layer 47, located in the middle, was selected as the reference layer, and the remaining layers were aligned to that layer to eliminate the impact of different acquisition times on the data; 2) realignment: images with translational head movement of more than 2 mm or a rotation angle greater than 2° were discarded from the NC; images with translational head movement of more than 3 mm or a rotation angle greater than 3° were discarded from the patients; and 3) normalization: after correction for head motion, the image space was standardized to the Montreal Neurological Institute (MNI) head anatomy template and resampled with 3 × 3 × 3 mm voxels.^4^ Filtering: slow5 (0.01–0.027 Hz), slow4 (0.027–0.073 Hz), slow3 (0.073–0.198 Hz), and slow2 (0.198–0.25 Hz) bandpass filters were used to eliminate the effects of physiological noise above and below these frequency bands, such as respiration and heartbeats.^5^, ^22^ Smoothing: after spatial standardization, the images were spatially smoothed using a Gaussian filter with a full‐width at half‐maximum (FWHM) of 6 mm to reduce the random noise of the images and improve the image signal‐to‐noise ratio.^6^ Nuisance covariate regression: physiological factors, white matter, cerebrospinal fluid, head movement, and other covariates were regressed out.

### Functional Connectivity Analysis and Multilayer Network Construction

#### MONOLAYER NETWORK CONSTRUCTION

An automated anatomical labeling (AAL) template was used to divide the cerebral cortex into 90 regions.^23^ Next, the time series of all voxels in each of these 90 brain regions were extracted and averaged. The Pearson correlation value between time series was used as the functional connection value between brain regions. To exclude false connections between nodes in the network, the minimum spanning tree (MST) was performed to binarize the matrix. Correlation matrices for each layer were binarized using the MST, which yields four 90 × 90 adjacency matrices.

#### MULTILAYER NETWORK CONSTRUCTION

**Figure 1:**
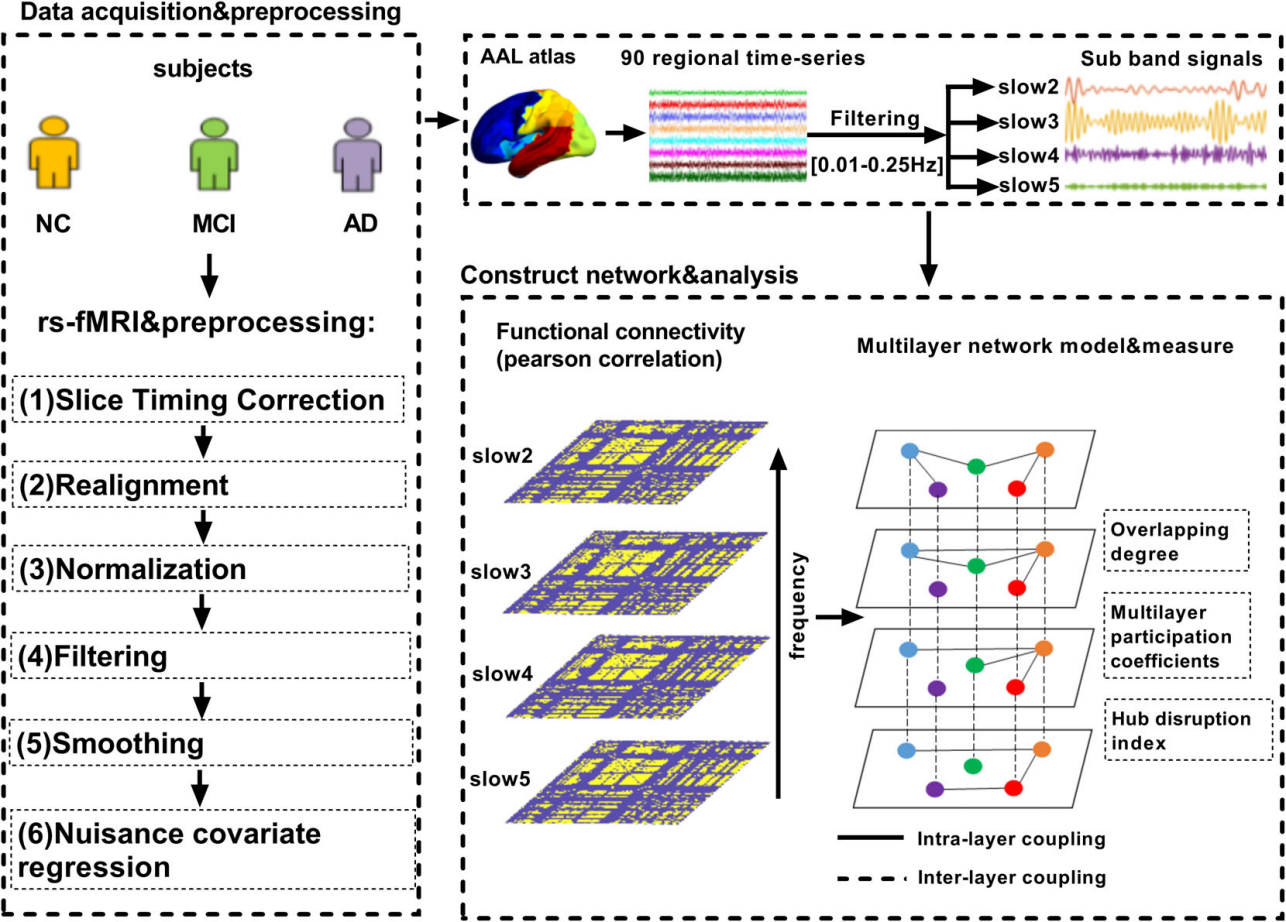
The flow chart of the experiment. The results of fMRI data preprocessing were first divided into 90 brain regions using the existing standard brain profile of the AAL template. Each brain region represents a node in the network. The brain physiological signal is decomposed into four frequency segments, each frequency band used as a layer. The intralayer connection is calculated by Pearson correlation, and the same nodes are connected between layers (regardless of the cross‐frequency coupling between different brain regions). Finally, some multilayer network measures were calculated.

For multilayer networks, there is a common representation method: the block adjacency matrix. A multilayer network with *f* layers can be expressed as:

$$\left[\begin{array}{ccc} A_{1} & \cdots & H_{1f}\\ \vdots & \ddots & \vdots \\ H_{f1} & \cdots & A_{f} \end{array}\right]$$

where *A*_*α*_ is the symmetric square matrix of layer *α*, 1 ≤ *α* ≤ *f* and *H*_*kl*_ refers to the connection matrix between layers k and l, 1 ≤ *k*, *l* ≤ *f*. Each layer has the same dimensions (N × N) as the adjacency matrix. In this study multilayer networks based on fMRI data are constructed by integrating four (slow2, slow3, slow4, slow5) frequency‐specific networks in which each layer shares the same set of nodes (*N* = 90) and the links in each layer are composed of functional connections within each frequency band. The same brain regions were connected on different layers to construct a four‐layer network based on resting‐state fMRI (Fig. 1).

### Multilayer Network Analysis

#### MULTILAYER CENTRALITY METRICS

The hub properties of multilayer networks were calculated. The importance of a region relative to the overall functional connectivity of the brain can be quantified by centrality metrics. The degree in a single‐layer network was extended to a multilayer network as a measure called the overlapping degree of nodes.^24^ The degree of node i in layer *α* can be defined in the following formula:

(1) $$M_{i}^{\left[\propto \right]}=\sum_{j}a_{\mathrm{ij}}^{\left[\propto \right]}$$

where $a_{\mathrm{ij}}^{\left[\alpha \right]}$ represents the number of nodes connected to node i on layer *α*. The degree of node i in a multilayer network can be represented as a vector in the following formula:

(2) $$M_{i}=\left\{M_{i}^{\left[1\right]},\ldots ,M_{i}^{\left[f\right]}\right\},i=1,\ldots ,N\;$$

As a result, for node i, the overlapping degree can be represented in the following formula:

(3) $$O_{i}=\sum_{\propto }M_{i}^{\left[\propto \right]}$$

The same above steps were used to obtain the overlapping degree of each node. To compare multilayer networks of different sizes, we calculate the Z‐score and use the Z‐score to divide the nodes in the network into two types: hub nodes (*Z*_*i*_ > 1) and nonhub nodes (*Z*_*i*_ ≤ 1).

(4) $$Z_{i}=\frac{O_{i}–<o>}{\sigma }$$

where <*o*> is equal to the average overlapping degree of nodes in the network, while *σ* represents the corresponding standard deviation.

#### MULTILAYER PARTICIPATION COEFFICIENT

The multilayer participation coefficient (MPC) is an index to measure the global information processing capacity of multilayer networks. It is used to quantify the distribution of nodes' links at each layer. Generally, the more evenly distributed nodes are, the larger their MPC value will be. The MPC values belong to the range [0, 1], MPC = 0 means that the links of the node are only distributed at one layer, whereas MPC = 1 means that the number of links of the node is the same at each layer. Nodes with high MPC values are defined as interlayer core nodes, while nodes with low MPC values are defined as peripheral nodes. For node i, MPC is defined as:

(5) $${\mathrm{MPC}}_{i}=\frac{f}{f–1}\left[1–\sum_{\propto =1}^{f}{\left(\frac{O_{i}^{\left[\propto \right]}}{K_{i}}\right)}^{2}\right]$$

**Figure 2:**
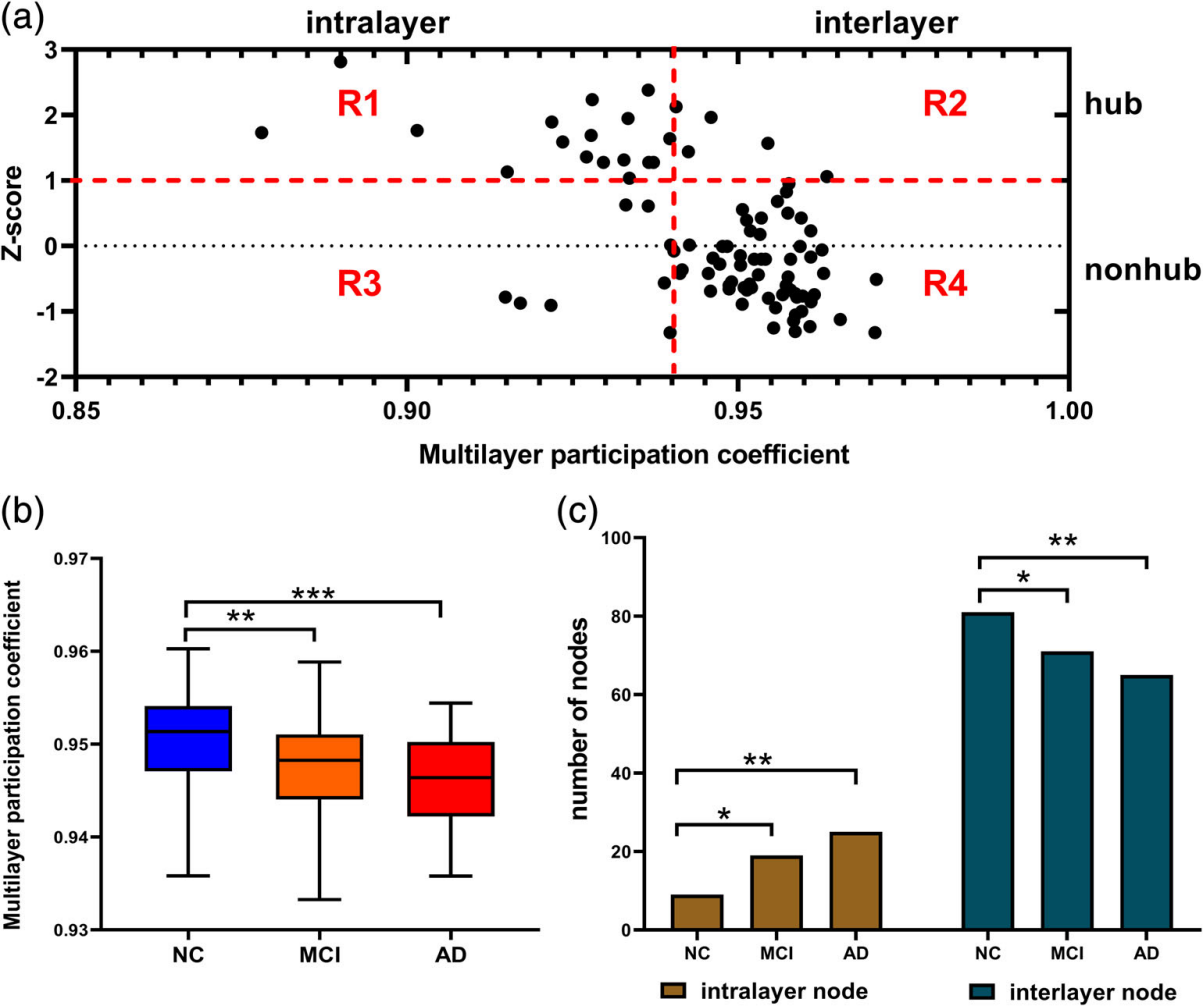
Central loss of the brain region in the multilayer network of MCI and AD patients. (**a**) Node type partitioning in the network. R1 = intralayer hub; R2 = interlayer hub; R3 = intralayer nonhub; R4 = interlayer nonhub. Nodes in R1 and R3 are collectively called intralayer nodes, while those in R2 and R4 are collectively called interlayer nodes. (**b**) The variation of the overall MPC of the three groups of subjects. (**c**) The trends in the numbers of interlayer nodes and intralayer nodes in the three groups. The differences are divided into three types according to the significance values: *(0.01 < *P* < 0.05),**(0.001 < *P* < 0.01), and ***(*P* = 0.000).

Since the Oi of the node represents its overall importance in terms of the number of incident edges and the MPC gives information on the cross‐layer distribution of the incident edges, so it can be classified by simultaneously looking at the MPC and Oi of the multilayer network nodes.^24^ Depending on the size of the MPC, it can divide the node into two types: interlayer node (*MPC*_*i*_ > 0.94) and intralayer node (*MPC*_*i*_ ≤ 0.94). Combined with the total overlapping degree, nodes in the network are divided into four types, as shown in Fig. 2a. There may be a situation in the network where some nodes belong to the interlayer hub in NC but transitioned into the intralayer hub in MCI or AD patients. These nodes that underwent this node‐type transformation were termed damaged hub nodes.

#### HUB DISRUPTION INDEX

To compare node centrality characteristics between patients and healthy controls, the hub disruption index (HDI) was calculated.^25^ To explore the abnormal pattern of hub nodes in the multilayer network of patients, this study calculated the HDI of MCI and AD based on MPC. After obtaining the MPC values of the individual subjects' brain network nodes, we subtracted the mean MPC values of the corresponding nodes in the healthy control group's brain network from these values. Then, taking the MPC values of the healthy control group's brain network nodes as a reference, the difference between the MPC values of the individual subjects' nodes and the average MPC values of the healthy control group's corresponding nodes were plotted as a scatterplot, and linear regression was used to fit straight lines to these data points; the slope (k) of the regression line was defined as the HDI.

#### Statistical Analysis

Before statistical analysis, it is necessary to check the distribution of data and homogeneity of variance of the population. This is the first prerequisite for the follow‐up difference analysis. In this experiment, three groups are independent of each other, each population obeys normal distribution, and has the same variance. In this study, multilayer network analysis was researched from a global perspective to a local perspective. From the overall level of the network, in order to research whether the cross‐frequency integration is normal in the patient's multilayer network, one‐way analysis of variance (ANOVA) was performed to examine differences in the overall MPC of the three groups. Moreover, the least significant difference (LSD) method that is most sensitive to variance was selected for the post‐hoc tests of ANOVA. Similarly, a Pearson chi‐squared two‐tailed test was performed to examine differences between the number of intralayer nodes and interlayer nodes to measure the changes in the relationship between the layers of the multilayer network as the disease progressed. The significance level for this study was set at *P* = 0.05.

From the node level, which brain area lesions caused the abnormality of the patient's multilayer network were researched. During the whole process, the changes of hub nodes in the network were given high attention. In addition, the nonparametric Spearman correlation coefficient *r* was used to find the abnormal distribution of hub nodes in the multilayer network and test the predictive ability of the multilayer network indicators on patients' cognitive impairment.

In order to avoid the Type I error in hypothesis testing, the results of intergroup difference analysis were corrected by the false discovery rate (FDR).^26^ FDR is often used for *P*‐value correction under multiple tests; what this means is the proportion of the number of false rejects in the number of rejected tests. Please refer to Appendix S1 for the specific algorithm of FDR.

## Results

### Loss of Interlayer Centrality

The difference between the three groups in the MPC value is shown in Fig. 2b. The results showed that the MPC of MCI patients decreased significantly compared with that of NC (*P* < 0.05), and the MPC of AD patients decreased significantly more that of the MCI patients (*P* < 0.05). Second, comparison of the number of different node types indicated that, as the disease progressed, the number of interlayer nodes decreased significantly, while the number of intralayer nodes increased significantly (Fig. 2c), and the MCI increase (*P* < 0.05) was less than AD (*P* < 0.05).

**Figure 3:**
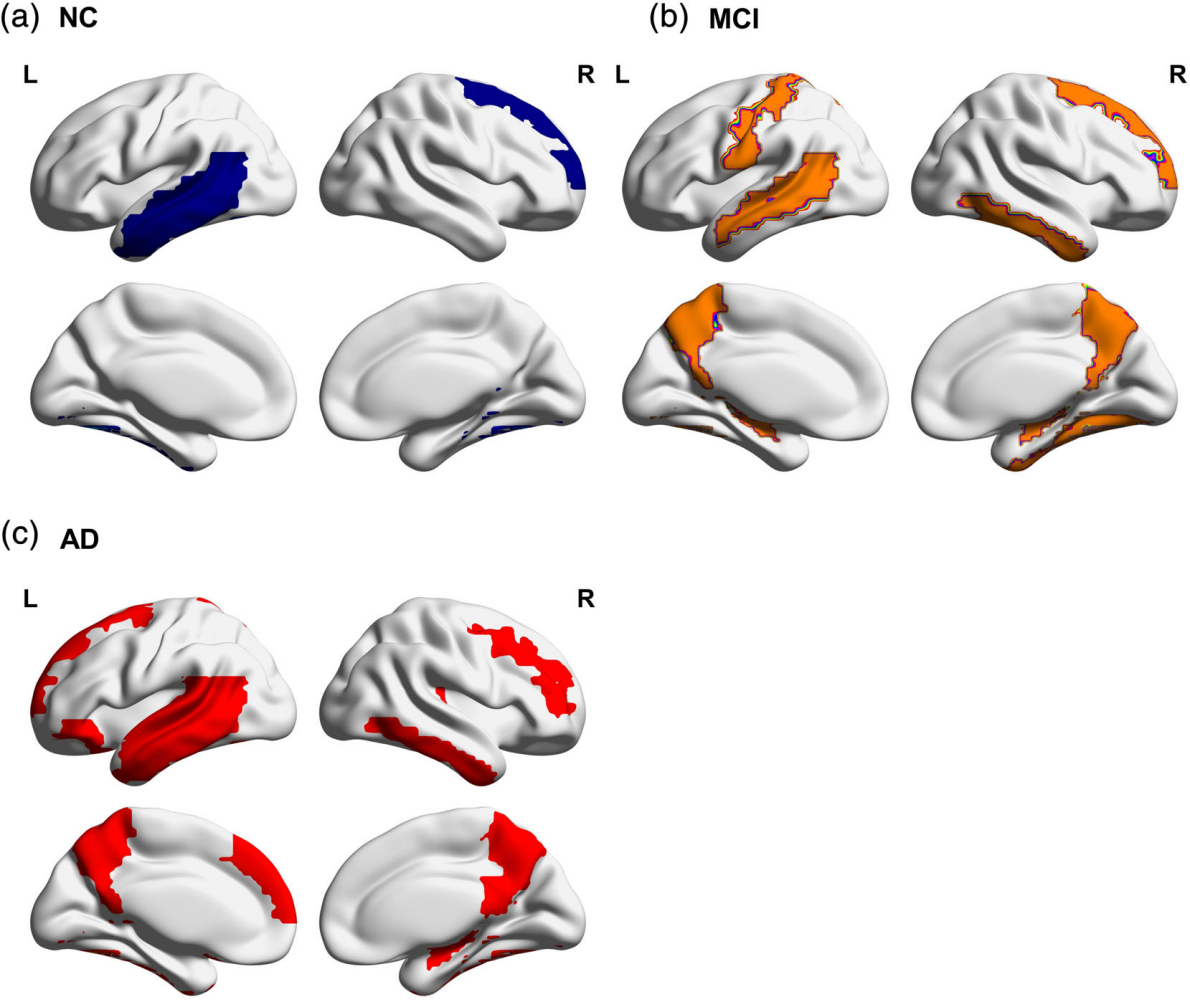
(**a–c**) The distribution of intralayer nodes in the NC, MCI, and AD groups.

For the sake of simplicity and intuition, this section shows only the intralayer nodes to avoid overcrowding the figures. The distribution of intralayer nodes in different subjects is shown in Table 2. Compared with NC，the intralayer nodes in the MCI group were mainly concentrated in the parietal lobe and the AD group are mainly concentrated in the frontal and temporal lobes (Fig. 3).

**TABLE 2:** Distribution of Intralayer Nodes of MCI and AD

Cortex	NC	MCI	AD
Frontal	Frontal_Sup [L/R] Frontal_Mid [L/R]	Precentral [L] Frontal_Sup [R] Frontal_Mid [L/R]	Precentral [L/R] Frontal_Sup [L/R] Frontal_Mid [R] Frontal_Inf_Oper [L] Frontal_Inf_Orb [L] Supp_Motor_Area [R] Frontal_Sup_Medial [L]
Parietal	Postcentral [L]	Postcentral [L/R] Parietal_Sup [L/R] Precuneus [L/R]	Postcentral_L Precuneus [L/R]
Temporal	Temporal_Mid [L/R] Temporal_Inf [L/R]	Temporal_Mid [L/R] Temporal_Inf [L/R]	Heschl [L/R] Temporal_Sup [L] Temporal_Pole_Sup [L] Temporal_Mid [L/R] Temporal_Pole_Mid [L] Temporal_Inf [L/R]
Limbic		Hippocampus [L/R]	Cingulum_Post [L/R] Hippocampus [L/R]
Occipital		Fusiform [L/R]	
Basal		Putamen [R]	

### Vulnerability of Central Nodes

**Figure 4:**
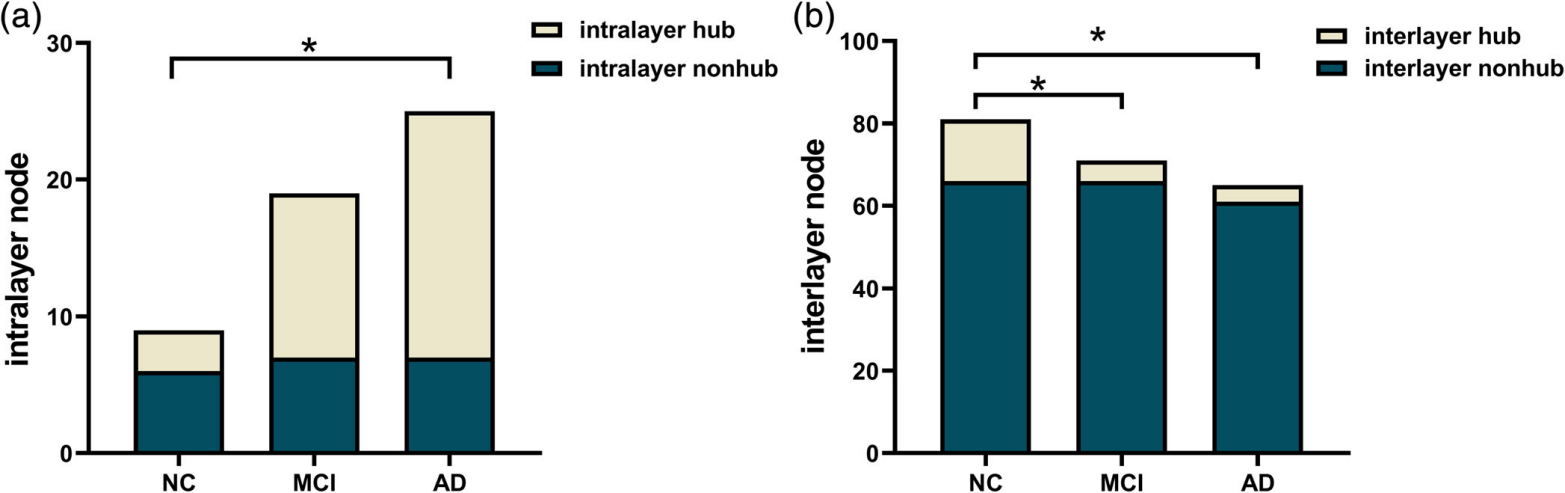
Comparison of the number of different node types in the multilayer networks of the three groups of subjects. (**a,b**) The proportions of hub and nonhub nodes among the intralayer nodes and interlayer nodes, respectively.

The proportion of hub and nonhub nodes among the intralayer nodes and interlayer nodes of different subjects was further analyzed (Fig. 4). The results of chi‐squared statistical tests show that the significant increase in the number of intralayer nodes from NC to AD is due to the significant increase in the number of intralayer hubs (*P* < 0.05). In the interlayer nodes, a significant decrease in the number of hub nodes in patients with MCI (*P* < 0.05) and AD (*P* < 0.05) as the disease worsened was observed.

**Figure 5:**
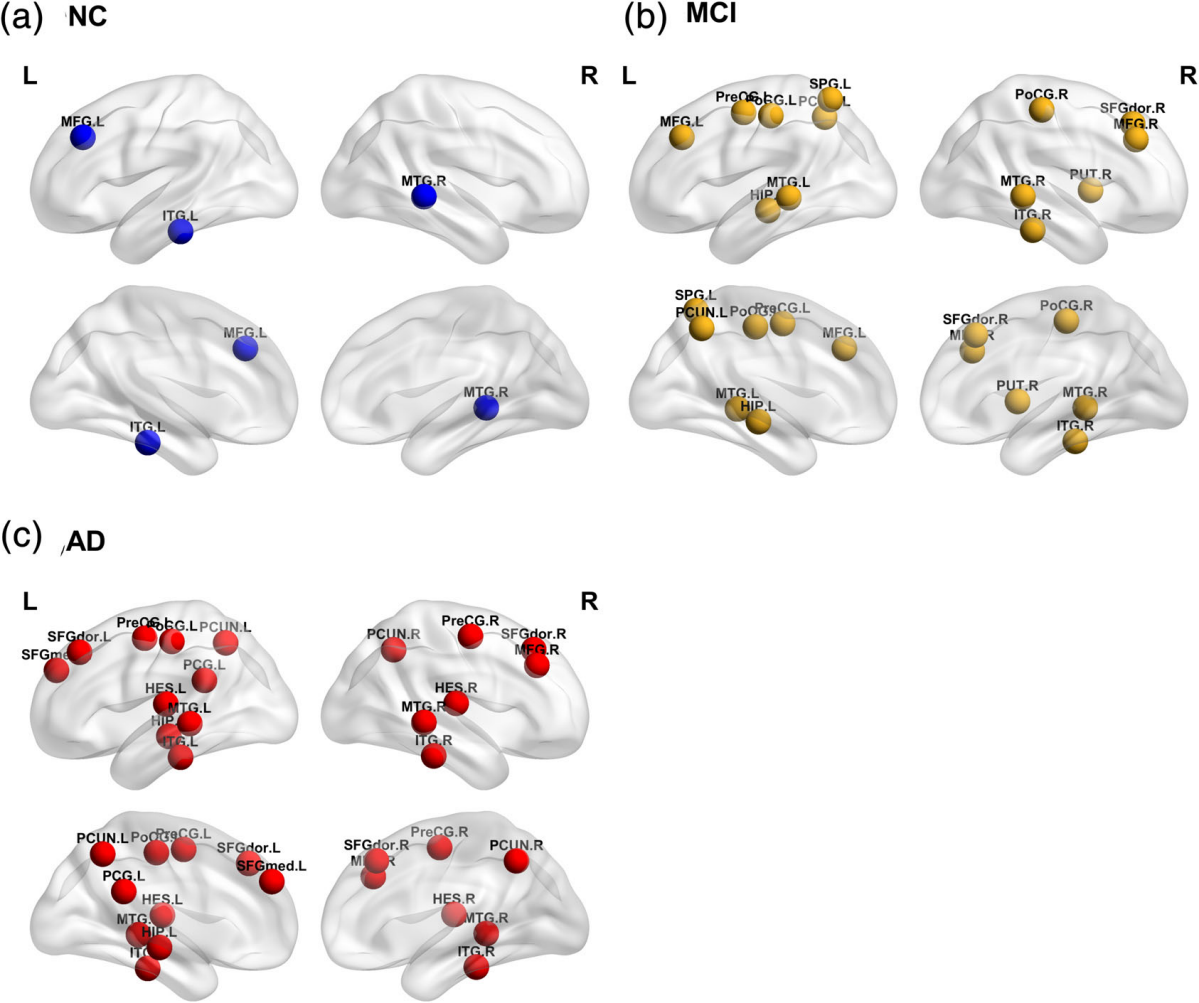
(**a–c**) The distribution of intralayer hubs in the NC, MCI, and AD groups.

As shown in Fig. 4, in the multilayer network of patients the hubs were preferentially damaged. Here, the distribution of hub nodes in the intralayer nodes of different subjects was further studied. As shown in Fig. 5a, the intralayer hub nodes in the NC include the left middle frontal gyrus (MFG.L), right middle temporal gyrus (MTG.R), and left inferior temporal gyrus (ITG.L). In Fig. 5b, the additional intralayer hub added to the MCI in addition to the original nodes in the NC are the left precentral gyrus (PreCG.L), right dorsal superior frontal gyrus (SFGdor.R), left hippocampus (HIP.L), left postcentral gyrus (PoCG.L), right postcentral gyrus (PoCG.R), left superior parietal gyrus (SPG.L), left precuneus (PCUN.L), right putamen (PUT.R), and left middle temporal gyrus (MTG.L). In Fig. 5c, the additional intralayer hubs added to the AD in addition to the original nodes in the NC are as follows: PreCG.L, right precentral gyrus (PreCG.R), left dorsal superior frontal gyrus (SFGdor.L), SFGdor.R, right middle frontal gyrus (MFG.R), left medial superior frontal gyrus (SFGmed.L), left posterior cingulate (PCG.L), HIP.L, PoCG.L, PCUN.L, right precuneus (PCUN.R), left Heschl's gyrus (HES.L), right Heschl's gyrus (HES.R), MTG.L, and right inferior temporal gyrus (ITG.R).

### Heterogeneity of Central Node Cross‐Layer Distribution

**Figure 6:**
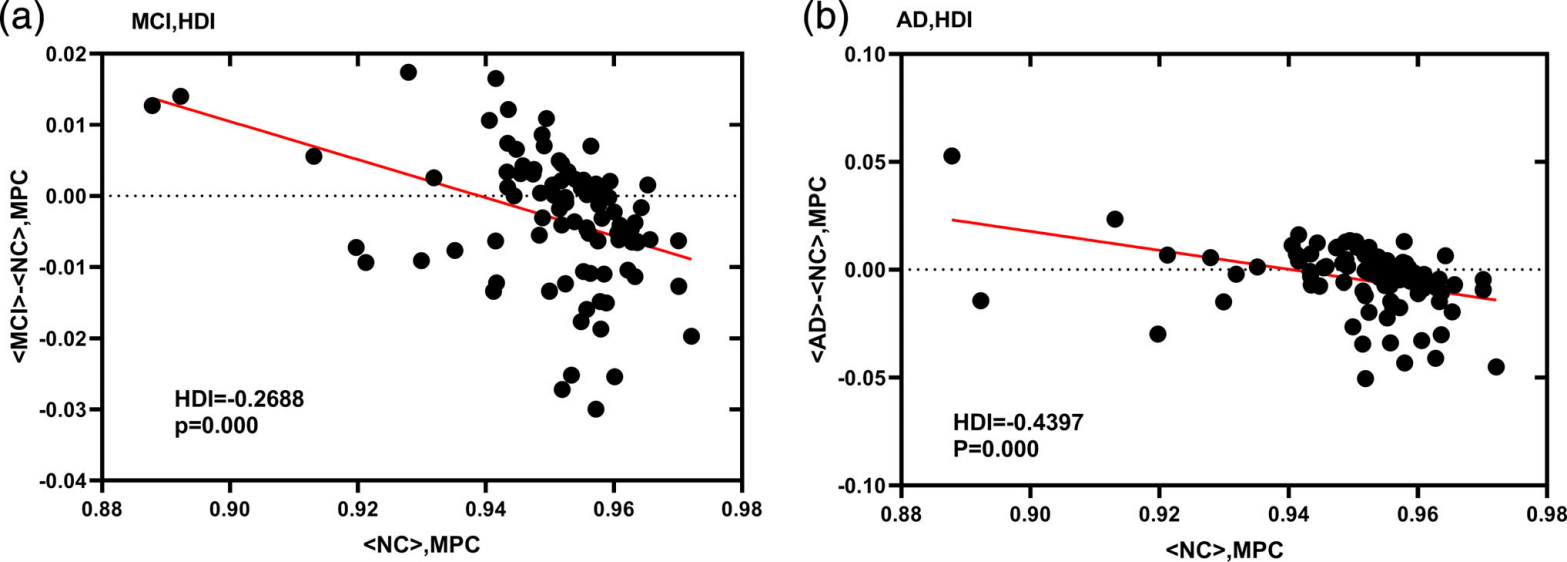
HDI of the functional network of MCI (**a**) and AD (**b**) patients. Both groups had very significant negative HDI, and the AD damage was more severe than MCI.

**Figure 7:**
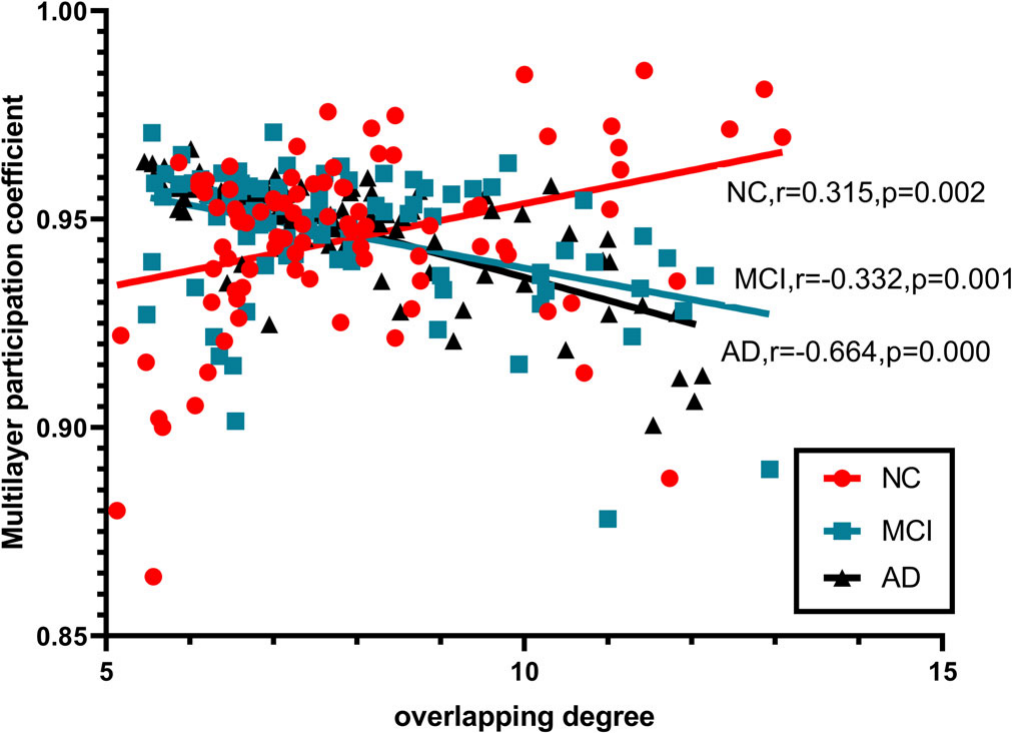
Heterogeneity in the cross‐layer distribution of brain regions in patients.

HDI was used to demonstrate the abnormal patterns of hub nodes. As shown in Fig. 6, AD patients (HDI = –0.4397, *P* < 0.05) showed a more negative HDI than MCI patients (HDI = –0.2688, *P* < 0.05). In addition, the result of the correlation between MPC and the overlapping degree (Oi) is shown in Fig. 7. The results showed that MPC was positively correlated with Oi in the NC group (Spearman's *r =* 0.315, *P* < 0.05). However, in MCI (Spearman's *r =* –0.3318, *P* < 0.05) and AD (Spearman's *r =* –0.6637, *P* < 0.05), MPC was negatively correlated with Oi.

### Changes in the Functional Roles of Central Nodes

The MPC of 12 brain regions in MCI patients was significantly lower than that in NC patients, as shown in Fig. S1a. These regions included: PreCG.L (P_FDR_ < 0.05), left inferior frontal gyrus, triangular part (IFGtriang.L) (P_FDR_ < 0.05), HIP.L (P_FDR_ < 0.05), right hippocampus (HIP.R) (P_FDR_ < 0.05), left fusiform gyrus (FFG.L) (P_FDR_ < 0.05), right fusiform gyrus (FFG.R) (P_FDR_ < 0.05), PoCG.R (P_FDR_ < 0.05), PCUN.L (P_FDR_ < 0.05), PCUN.R (P_FDR_ < 0.05), left lenticular nucleus (PUT.L) (P_FDR_ < 0.05), PUT.R (P_FDR_ < 0.05), HES.R (P_FDR_ < 0.05). Among these nodes, the damaged hub nodes included the PreCG.L (P_FDR_ < 0.05), HIP.L (P_FDR_ < 0.05) and PoCG.R (P_FDR_ < 0.05) (Fig. S1c). The MPC of 16 brain regions in AD patients was significantly lower than that in NC patients, as shown in Fig. S1b. These regions included: PreCG.L (P_FDR_ < 0.05), PreCG.R (P_FDR_ < 0.05), left inferior frontal gyrus, opercular part (IFGoperc.L) (P_FDR_ < 0.05), right supplementary motor area (SMA.R) (P_FDR_ < 0.05), SFGmed.L (P_FDR_ < 0.05), PCG.L (P_FDR_ < 0.05), right posterior cingulate gyrus (PCG.R) (P_FDR_ < 0.05), HIP.L (P_FDR_ < 0.05), HIP.R (P_FDR_ < 0.05), PCUN.L (P_FDR_ < 0.05), PCUN.R (P_FDR_ < 0.05, left caudate nucleus (CAU.L) (P_FDR_ < 0.05), HES.L (P_FDR_ < 0.05), HES.R (P_FDR_ < 0.05), left temporal pole: superior temporal gyrus (TPOsup.L) (P_FDR_ < 0.05), left temporal pole: middle temporal gyrus (TPOmid.L) (P_FDR_ < 0.05). In the AD, the damaged hub nodes included the PreCG.L (P_FDR_ < 0.05), SFGmed.L (P_FDR_ < 0.05), PCG.L (P_FDR_ < 0.05), HIP.L (P_FDR_ < 0.05), and PCUN.L (P_FDR_ < 0.05) (Fig. S1d).

### *Correlation Between Multilayer Centrality Metrics and* *Mini‐Mental* *State Examination Scores*

The MPC was significantly correlated with the clinical symptoms of AD and MCI (Fig. S2). Moreover, the correlation between MPC and the Mini‐Mental State Examination (MMSE) score was stronger in AD patients (Spearman's *r =* 0.526, *P* < 0.05) than in MCI patients (Spearman's *r =* 0.229, *P* < 0.05).The specific relevant brain regions were located (Table 3). In these brain regions, the MPC values of some damaged hub nodes in MCI (PreCG.L: Spearman's *r =* 0.232, P_FDR_ < 0.05) and AD (PreCG.L: Spearman's *r =* 0.604, P_FDR_ < 0.05; PCG.L: Spearman's *r =* 0.544, P_FDR_ < 0.05; HIP.L: Spearman's *r =* 0.507, P_FDR_ < 0.05; SFGmed.L: Spearman's *r =* 0.391, P_FDR_ < 0.05) were significantly correlated with MMSE scores.

**TABLE 3:** Abnormal Cognitive Function of MCI and AD Patients Is Positively Correlated With the Vulnerability of Central Regions

Group	Correlation	Rank	ROI label	Cortex	R coeff	P_FDR_‐value
MCI	MPC‐MMSE	1	PCUN.L	Parietal	0.417	P_FDR_ < 0.05
		2	SPG.L	Parietal	0.296	P_FDR_ < 0.05
		3	HES.R	Temporal	0.284	P_FDR_ < 0.05
		4	IPL.R	Parietal	0.258	P_FDR_ < 0.05
		5	MFG.L	Frontal	0.257	P_FDR_ < 0.05
		6	**PreCG.L**	Frontal	0.232	P_FDR_ < 0.05
AD	MPC‐MMSE	1	HES.R	Temporal	0.782	P_FDR_ < 0.05
		2	SFGdor.R	Frontal	0.713	P_FDR_ < 0.05
		3	**PreCG.L**	Frontal	0.604	P_FDR_ < 0.05
		4	**PCG.L**	Limbic	0.544	P_FDR_ < 0.05
		5	**HIP.L**	Limbic	0.507	P_FDR_ < 0.05
		6	HES.L	Temporal	0.474	P_FDR_ < 0.05
		7	PCUN.R	Parietal	0.450	P_FDR_ < 0.05
		8	ORBmid.R	Frontal	0.405	P_FDR_ < 0.05
		9	**SFGmed.L**	Frontal	0.391	P_FDR_ < 0.05

The entries are sorted according to the obtained *P* values. The brain areas written in bold in the table belong to the hub regions that were significantly damaged in the corresponding subjects.

## Discussion

The results of this research found that the process of atrophy in AD can lead to impaired cross‐frequency integration in patient multilayer networks, which is mainly reflected in a decrease in interlayer interaction. With the deterioration of the disease, the number of intralayer nodes increases, indicating that a large number of nodes tend to work in the layer. Among these nodes, the proportion of hub nodes increases, indicating that hub nodes are more vulnerable to damage than nonhub nodes are. Moreover, these vulnerable hubs were correlated with the cognitive scores of patients with MCI and AD, indicating that the cognitive function of patients was related to the vulnerability of central regions in the multilayer network.

Compared with NC, MCI and AD patients had impaired cross‐frequency integration. This can be explained by an evident decrease in the overall MPC of the network and an evident increase in the number of intralayer nodes. As mentioned above, nodes with high MPC values are considered hubs between layers, as they facilitate information exchange between different layers and play a crucial role in communication between different layers. Therefore, during disease progression the decrease in MPC in a large number of brain regions indicates that the ability of these brain regions to communicate between layers is gradually lost, and their function in promoting the spread of information across frequency bands is reduced.

Among MCI patients, this loss of interfrequency centrality is mainly concentrated with the parietal region, indicating that the brain regions with obvious impaired interlayer information exchange ability are mainly distributed in the parietal lobe. Parietal dysfunction is an important feature of early AD.^27^ A recent neuroimaging study using structural, functional and metabolic imaging methods has demonstrated abnormal brain changes in patients with MCI or prodromal AD.^28^ The results show that regions within the parietal lobe undergo a degenerative process and that the parietal lobe is involved in the early stages of AD. The results of this study further supported the conclusion that parietal function is abnormal in early AD, and parietal function is abnormal in multilayer network interlayer interactions.

This study also found that AD patients have a greater reduction than MCI patients in the global propensity for cross‐layer information exchange. During AD, it is evident that a large number of intralayer nodes are mainly concentrated in the frontal and temporal lobes, indicating that the brain regions with impaired interlayer information exchange ability are mainly distributed in the frontal and temporal lobe regions. Damage in these regions can lead to inadequate attention, awareness, and planning. A pathological study suggests that both clinical and pathological progression of AD is characterized by persistent loss of pyramidal cells in the frontal and temporal cortex.^29^ Frontal and temporal cortical atrophy is associated with mental symptoms in AD patients.^30^, ^31^

The above conclusions have shown that, during disease progression, a large number of hub nodes begin to work within the layer, resulting in a significant increase in the number of nodes in the layer. Therefore, it can be concluded that the hub nodes in the multilayer network are preferentially destroyed. The degree of damage in MCI patients is intermediate between NC and AD. The HDI results further confirm this conclusion. The hub nodes in the network face a high risk of damage in AD and provide the easiest explanation for the pathological mechanisms of AD. However, it is not clear whether the central region in AD is also destroyed in multilayer networks. It is clearly of great significance to explore the characteristics of central nodes in patients' multilayer networks. The results of this study not only identified the vulnerability of central nodes in the patient's multilayer network but also showed that the degree of damage to central nodes increased with the progression of the disease.

Moreover, this study found that there was heterogeneity in the cross‐layer distribution of central nodes in the multilayer network of MCI and AD patients compared to NC. The MPC is used to quantify the degree of participation of each node in each layer. Previous study has shown that two nodes with the same overlapping degree (Oi) may have great heterogeneity in their MPC values, which means that the roles of nodes in different layers are also different.^24^ The negative correlation between MPC and Oi indicates that the degree of the hub region in patients is unevenly distributed at all levels. This heterogeneity is more pronounced in AD than in MCI. This indicates that the hub region has different connection modes on different layers and plays different roles in different frequency bands. At the same time, it also reflects that the topology of different frequency bands is not consistent and that these bands are not independent. In the process of cognition, each brain region has different functions in different frequency bands, and optimal cognitive function may require the interaction of each frequency band.^18^

In this study we found that the main damaged hub regions in the multilayer network of patients. The node types of these nodes changed from interlayer hub in NC to intralayer hub in patients, with a loss of interlayer centrality. This study also detected a positive correlation between these vulnerable hub nodes and the MMSE scores of patients with MCI and AD, indicating that cognitive dysfunction in patients is associated with the vulnerability of hub regions in the multilayer network.

A feature of the brain map is the presence of nodes with a central role in the network, ie, “hubs.” The presence of hubs in the network makes the graph more resilient to random attacks on nodes or edges.^32^ However, hubs are also the weakest links in the network.^33^ Studies have shown that hubs in the network may be selectively vulnerable in AD patients.^34^, ^35^ Hub loss in AD may be explained by atrophy in specific regions that are known to be compromised. The areas of cortical atrophy in AD are mainly memory‐related structures, such as the hippocampus and other medial temporal lobe regions, as well as the precuneus, cingulate, and prefrontal regions.^36^, ^37^ A study has reported a decreased centrality of the posterior cingulate and medial temporal structures, which also indicated hub loss.^38^ Moreover, lower local centrality was associated with greater cognitive decline.^39^

A study of gray matter volume in early AD suggests that the left hippocampus atrophy is a useful feature for the early diagnosis of AD.^21^ In this study it also found that the left hippocampus was selectively vulnerable in MCI and AD patients' multilayer networks, and damage to this region was more severe in AD than in MCI. The posterior cingulate and precuneus are the hubs of the default‐mode network (DMN), vulnerable to functional connectivity interruptions in MCI and AD.^40^ This study also found abnormal patterns of these regions in patients' multilayer networks, which was more impaired in AD than in MCI. A previous study confirmed that the precentral gyrus is a connecting hub in human brain networks.^38^ Similarly, its centrality in AD patient's multilayer network was also disrupted. Previous research has found that the precentral gyrus shows significant gray matter volume reduction in patients with amnestic MCI, indicating that this region is atrophied in early AD.^41^ In this study the progression from MCI to AD is also accompanied by the gradual development of significant anomalies in multilayer networks. In addition, an anomaly of the postcentral gyrus in multilayer networks of MCI was found. Taken together, the evidence suggests that integrating functional networks with different frequencies into a multilayer network framework can provide additional information that traditional network methods miss. This study not only demonstrates the abnormal decline of cross‐frequency integration in patients with MCI and AD, but also determine which hub nodes in the multilayer network are especially vulnerable.

### Limitations

1) In multilayer networks, the estimation and allocation of interlayer links still needs further study. The strength of the interlayer connection is parametric and therefore arbitrary, and the biological interpretation of its representative remains to be elucidated. 2) Studies have shown that cross‐frequency coupling is a mechanism of interaction between different frequencies. Interlayer interactions between functional networks established in this study may reflect the corresponding mechanisms of fMRI signals. Nevertheless, the electrophysiological significance represented by this interlayer interaction is not clear, and the fMRI neural mechanism represented by different frequency components is not clear. This study only considered the interaction of the corresponding brain regions between different layers, but this is only the case in a multilayer network. In the future, we can consider researching the multilayer network of patients in more complex situations, such as considering the cross‐frequency coupling between different brain regions. 3) In this study only single centrality (the overlapping degree) was used to define hub regions. However, the number of hub regions may be influenced by the type of centrality measure. In future research, we should add other centrality measures. 4) The experimental results are based on the small ADNI dataset, which may lead to a lack of universality of the results. This method will be applied to a larger number of samples in future work. 5) Due to the limitation of data sources, the data used in this experiment were obtained by the conventional slow sampling rate (TR = 3). Although we have tried to eliminate noise during data preprocessing, spectrum leakage, and aliasing due to low sampling may still cause the BOLD signal to be affected by physiological noise such as breathing and heartbeat.

## Conclusion

This study analyzed the multilayer frequency brain networks of MCI and AD and found that there was an evident deficit in cross‐frequency integration in the multilayer networks of these patients compared to those of healthy controls. The reason for this anomaly is the loss of centrality between the frequencies of the hub nodes in the network, and these vulnerable hubs are associated with patients' cognitive scores. This research indicated that the multilayer network provides an effective framework for integrating networks of different frequencies, and this method can be used to identify the interfrequency neural mechanisms of AD.

## Supporting information


**Fig S1** Damaged hub regions in the multilayer networks of MCI and AD patients. The brain regions with significantly reduced MPC in MCI and AD patients compared to NC are shown in parts (a) and (b), respectively. (c) and (d) represent damaged hub nodes in MCI and AD patients, respectively. These nodes correspond to the brighter nodes in (a) and (b), respectively.

**Fig S2** Correlation between MPC and cognitive scores in (a) MCI and (b) AD patients. Both groups had significant positive correlations, and the correlations were stronger in AD than in MCI.

**Appendix** **S1** Supporting information.
