# Supplementary figures and images for: Deficit of Cross‐Frequency Integration in Mild Cognitive Impairment and Alzheimer's Disease: A Multilayer Network Approach
Source: J Magn Reson Imaging. 2020 Nov 26;53(5):1387–98. doi: 10.1002/jmri.27453 (PMC8247269; doi:10.1002/jmri.27453)

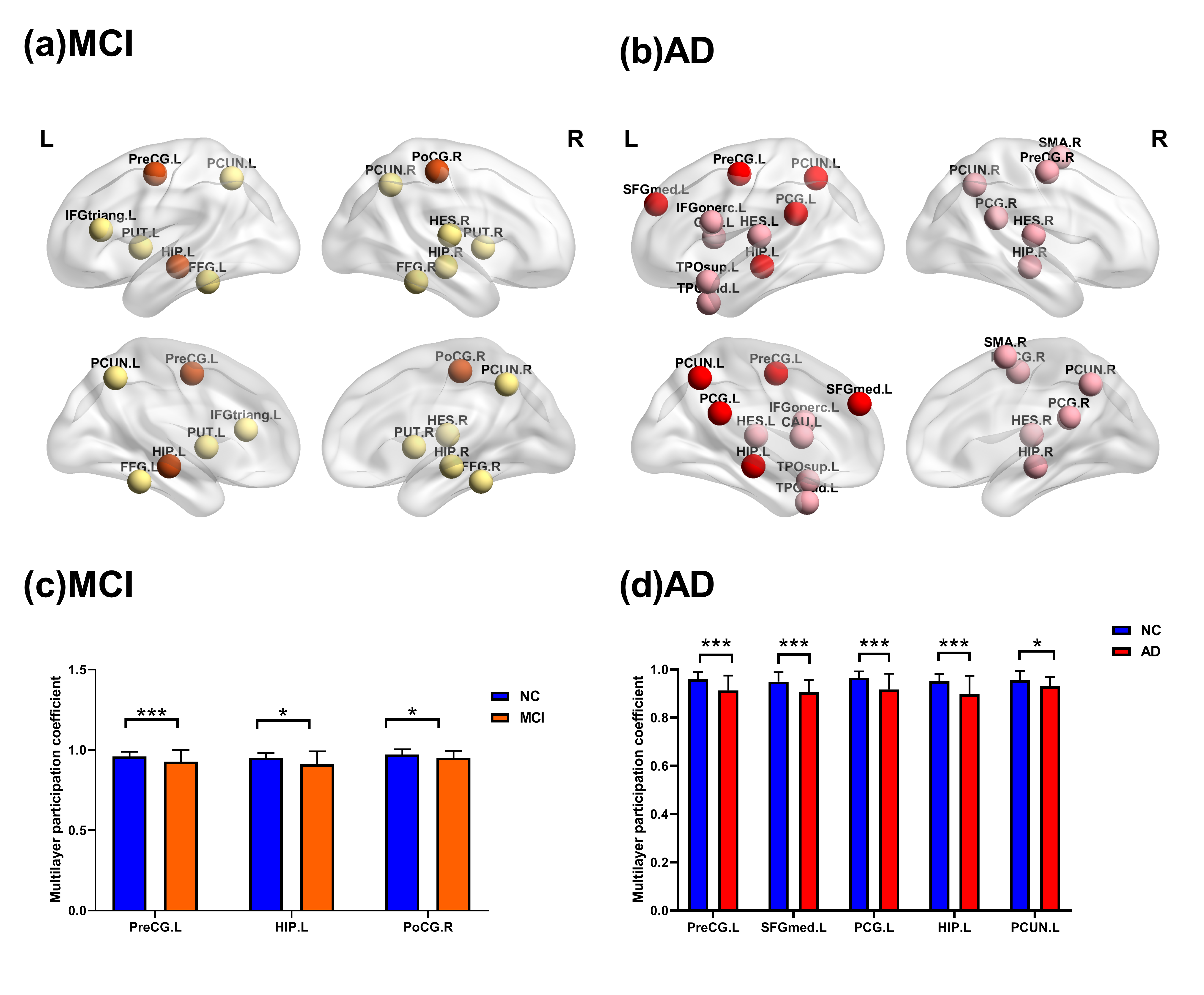

Supplement: Supplementary file 1 — Fig S1 Damaged hub regions in the multilayer networks of MCI and AD patients. The brain regions with significantly reduced MPC in MCI and AD patients compared to NC are shown in parts (a) and (b), respectively. (c) and (d) represent damaged hub nodes in MCI and AD patients, respectively. These nodes correspond to the brighter nodes in (a) and (b), respectively. [file JMRI-53-1387-s001.tif]

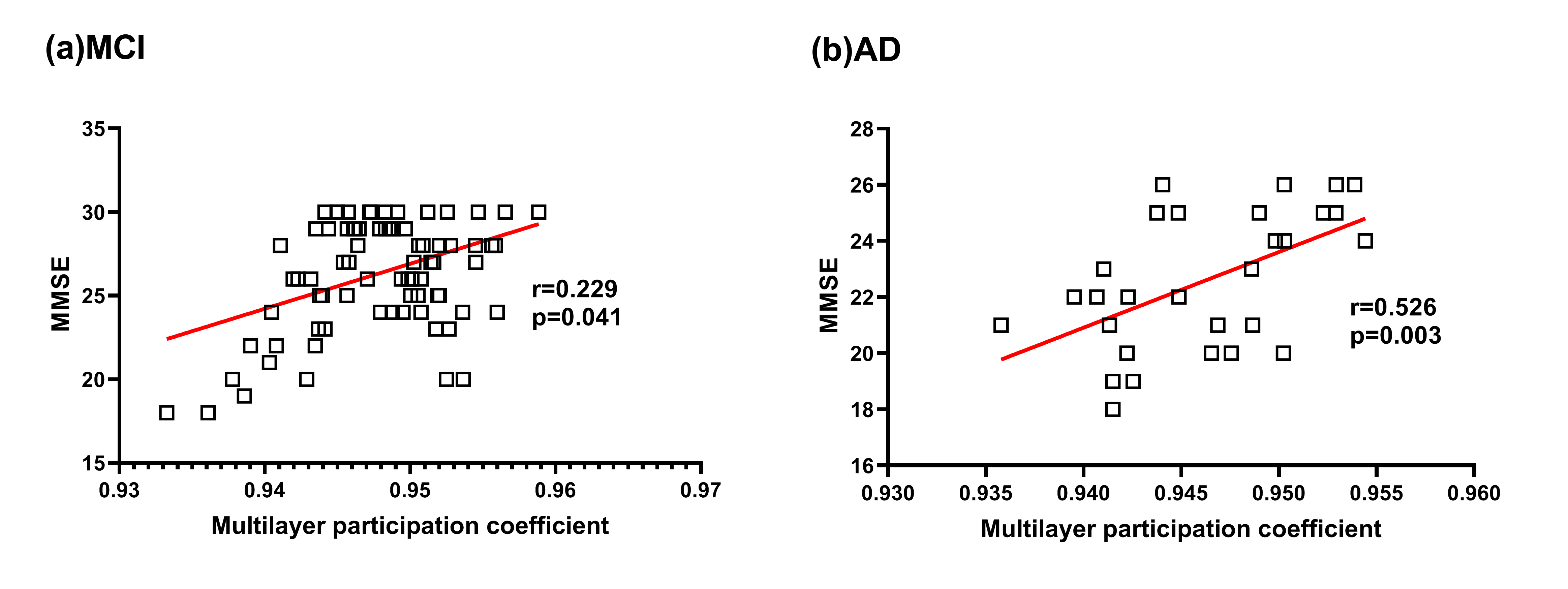

Supplement: Supplementary file 2 — Fig S2 Correlation between MPC and cognitive scores in (a) MCI and (b) AD patients. Both groups had significant positive correlations, and the correlations were stronger in AD than in MCI. [file JMRI-53-1387-s003.tif]
