# Supplementary material for: Deficit of Cross‐Frequency Integration in Mild Cognitive Impairment and Alzheimer's Disease: A Multilayer Network Approach
Source: J Magn Reson Imaging. 2020 Nov 26;53(5):1387–98. doi: 10.1002/jmri.27453 (PMC8247269; doi:10.1002/jmri.27453)
Supplement: Supplementary file 3 — Appendix S1 Supporting information. [file JMRI-53-1387-s002.docx]

**Supplementary materials for review**

**“Deficit of cross-frequency integration in mild cognitive impairment and Alzheimer's disease: A multilayer network approach”**

1. **Supplementary method**
   1. **False discovery rate (FDR)**
   2. **Temporal signal-to-noise(SNR)**
   3. **Realignment**
2. **Supplementary Figs**

**2.1 Supplementary Fig 1**

**2.2 Supplementary Fig 2**

**2.3 Supplementary Fig 3**

**2.4 Supplementary Fig 4**

**2.5 Supplementary Fig 5**

**2.6 Supplementary Fig 6**

**2.7 Supplementary Fig 7**

**2.8 Supplementary Fig 8**

**2.9 Supplementary Fig 9**

**2.10 Supplementary Fig 10**

**2.11 Supplementary Fig 11**

**References**

1. **Supplementary method**

**1.1 False discovery rate (FDR)**

Benjamini and Hochberg first proposed the concept of false discovery rate (FDR) in 1995(1). Its meaning is the expected value of the ratio of the number of false rejections (rejected null hypotheses) to the number of rejected null hypotheses. The domain value of P value was determined by controlling FDR. Suppose the results of the experiment found R brain regions with differential expression, among which S brain regions were truly differentially expressed, and V brain regions with no differential expression were false positive.In practice it is expected that the error ratio Q = V/R should on average not exceed a preset value (such as 0.05), which is statistically equivalent to controlling FDR at less than 5%.A formula can be used to intuitively express the meaning of FDR.

$$FDR=\frac{FP}{FP+TP}=1-PPV$$

TP (True Positive) is when a Positive result is detected on a Positive test sample.FP (False Positive) is the Positive result on a negative test sample. PPV is a Positive Predictive Value, which is often called Precision.

In this paper, we use Benjamini-Hochberg algorithm to calculate FDR, where n is the total number of samples, p value represents the significance value of each sample after comparison between groups, and the output (FDR value) of the algorithm represents the result after FDR correction for the original p values, namely the real p value[37].The significance level used in this paper was 0.05.The results of the algorithm were analyzed, and only the samples with FDR value less than 0.05 had real statistical difference.The steps are as follows:

Step1: Sort the p values from small to large, and mark the upper serial numbers 1-n;

Step2: The maximum FDR(It is the nth bit without considering repetition) is equal to the maximum p value;

Step3: For n-1 bit FDR, take smaller values of the following two:

(1) The FDR value calculated in the previous step (the nth bit);

(2) p*n/(n-1)

step4:Continuous iteration Step3 (n-2, n-3...), until the FDR corresponding to the minimum p value is calculated.

**1.2 Temporal signal-to-noise (SNR)**

The signal to noise ratio (SNR) is one of the important measures of the performance of a magnetic resonance imaging (MRI) system (2-6). It is often used to evaluate image quality. The SNR is defined as the ratio of the mean signal of a region of interest (ROI) to its standard deviation (7-11).The BOLD signal time series is positive without removing the covariates. But after regression of signals such as head movement, white matter, and cerebrospinal fluid, negative values were unavoidable. After the covariates are removed by linear regression, the remaining residuals are used for subsequent processing, and the mean value of residuals is 0. As a result, the average signal strength calculated under the whole time series is basically equal to 0, so the SNR cannot be calculated. Therefore, the calculation of SNR in this paper is based on the signal strength obtained in the first five steps of data preprocessing. The specific calculation formula is as follows:

$$SNR=\frac{S\_MEAN}{SD}$$

where S_MEAN represents the mean signal strength in the region of interest and SD represents the standard deviation of signal strength in this region of interest.

According to the evaluation standard of SNR, the SNR of MRI system of 1.5T or above should not be less than 180;The SNR of MRI system at 1.0T should be no less than 160;The SNR of the MRI system at 0.9t or below should not be less than 100.The results show that the SNR calculated at each frequency band meets the standard requirements, which indicates that the noise in the data is less and the data quality is higher.

The signal strength of the ROI of the three groups is not significant in the four sub-bands (slow2: 0.198-0.25Hz, slow3: 0.073-0.198Hz, slow4: 0.027-0.073Hz, slow5: 0.01-0.027Hz). The results are shown in the Supplementary Fig1.

**1.3 Realignment**

Even when the subjects' heads were well fixed, they would involuntarily move their heads slightly, which was particularly evident in the fMRI experiment. In the process of data preprocessing, we use the Realignment method to align each frame of an experimental sequence with the first frame of the sequence according to a certain algorithm to correct the head movement. If the signal is within the allowable range of head movement, certain algorithms can be used to correct the signal so that it is close to the true value; if it exceeds this specified range, this set of data must be eliminated. The range of head movement set in this experiment is: Normal control(translation≤2.0mm and rotation≤2.0degree) and patients(translation≤3.0mm and rotation≤3.0degree). According to the RealignParameter folder after Realignment, we can in ExcludeSubjectsAccordingToMaxHeadMotion.txt file to read head moving data. The following shows the excluded data in each group and the corresponding head motion curve of these subjects. In addition, we also listed the head movement information of several eligible subjects to show the results of our Realignment. Therefore, in our experiment, there were no subjects with very serious motion artifacts.

(1)NC:

Excluding Criteria: 2.0mm and 2.0 degree in max head motion

031_S_4021(Supplementary Fig2)

031_S_4496(Supplementary Fig3)

100_S_5246(Supplementary Fig4)

(2)MCI:

Excluding Criteria: 3.0mm and 3.0 degree in max head motion

None

(3)AD:

Excluding Criteria: 3.0mm and 3.0 degree in max head motion

130_S_5231(Supplementary Fig5)

For the eligible Normal control(NC), three subjects were randomly selected to display their head motion curves(Supplementary Fig6, Supplementary Fig7, Supplementary Fig8). For the eligible Mild cognitive impairment(MCI) and Alzheimer's disease(AD), three subjects were randomly selected to display their head motion curves(Supplementary Fig9, Supplementary Fig10, Supplementary Fig11).

1. **Supplementary Figs**

**2.1 Supplementary Fig 1**


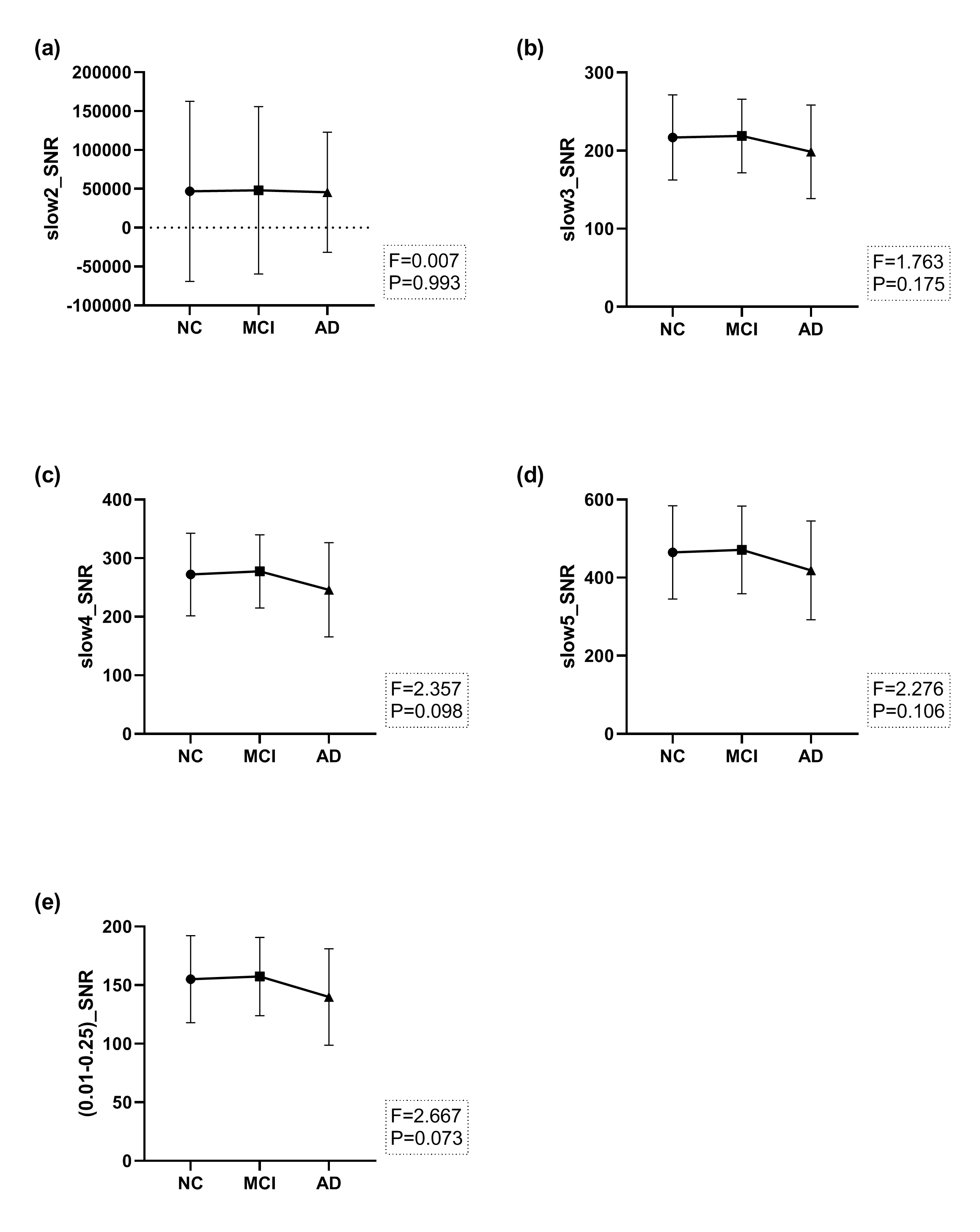


**Supplementary Fig1.** (a)-(d) represent the comparison of signal strength of three groups in four sub-bands.

**2.2Supplementary Fig 2**


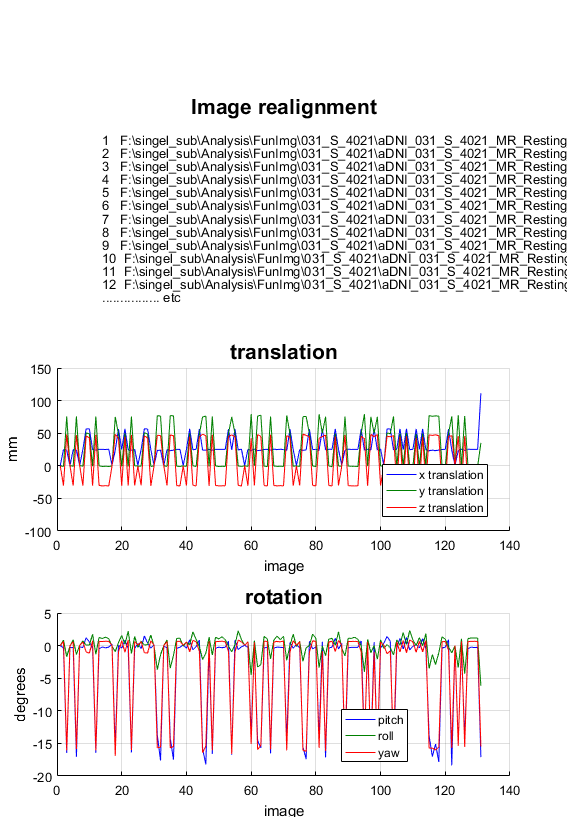


**2.3 Supplementary Fig 3**


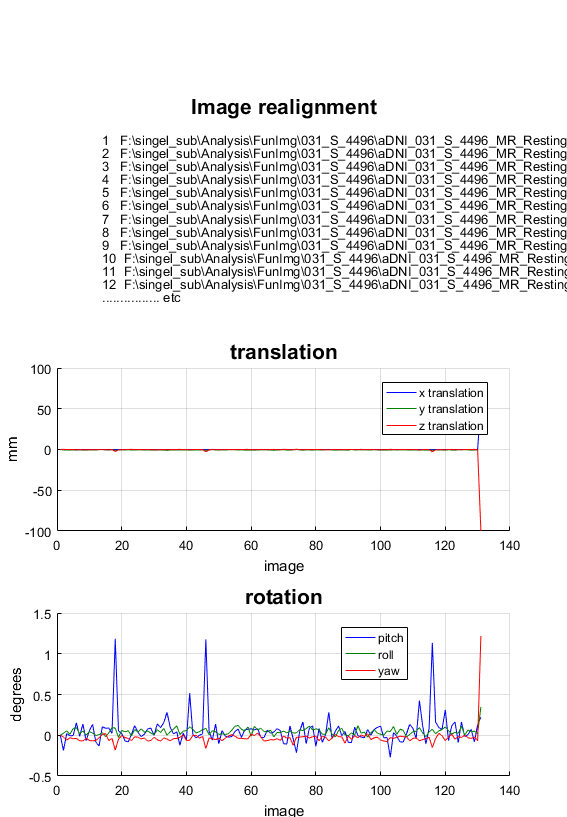


**2.4 Supplementary Fig4**


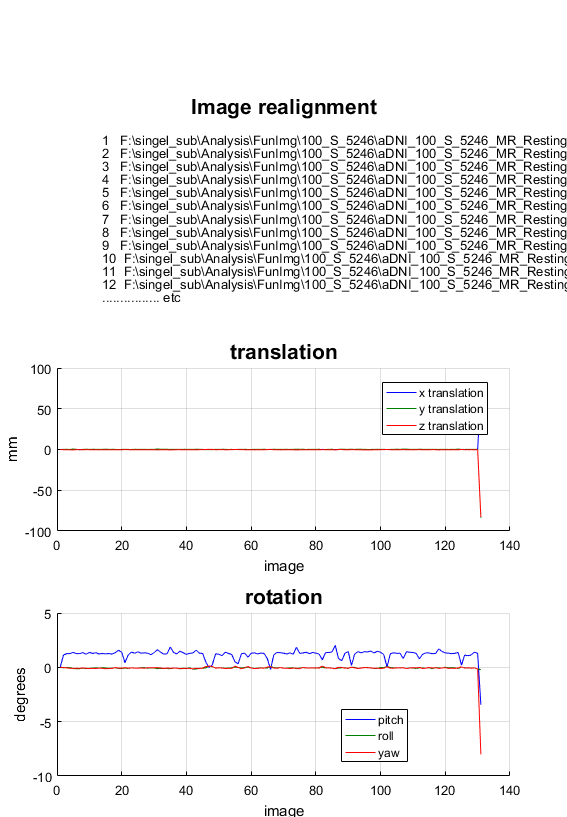


**2.5 Supplementary Fig 5**


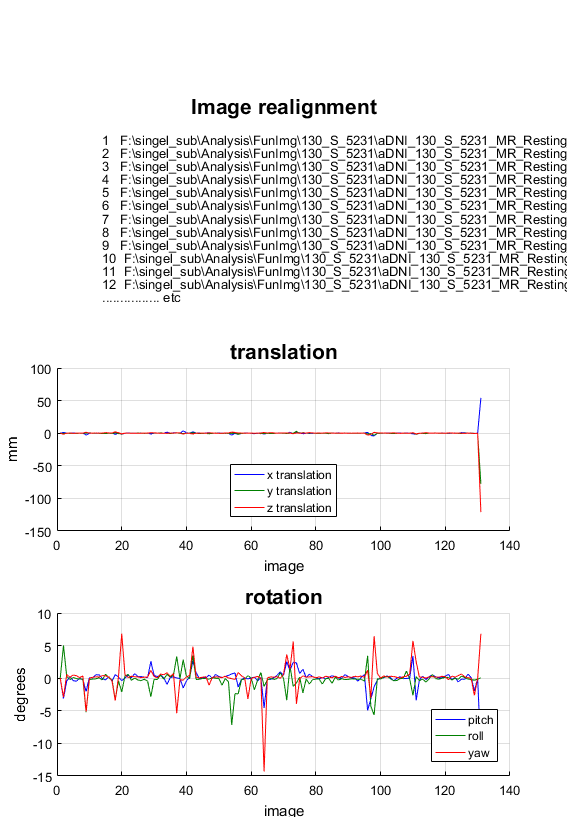


**2.6 Supplementary Fig 6**


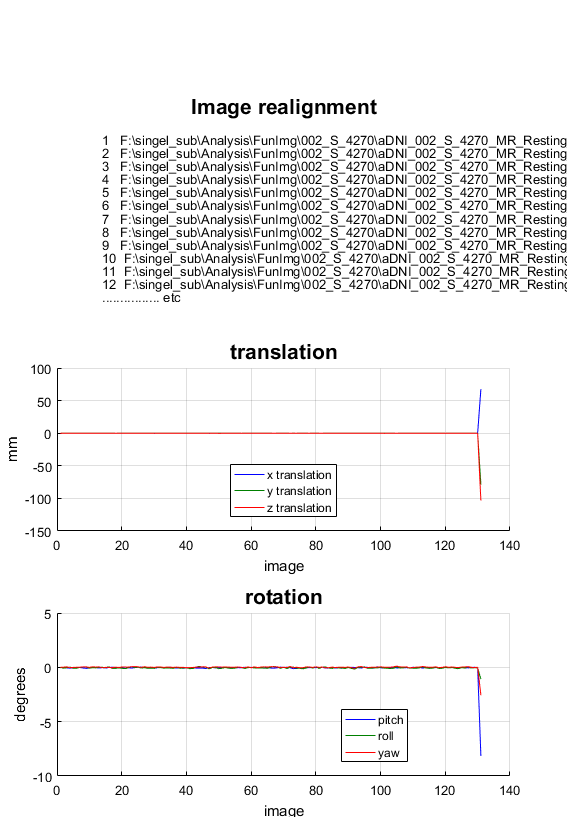


**2.7 Supplementary Fig 7**


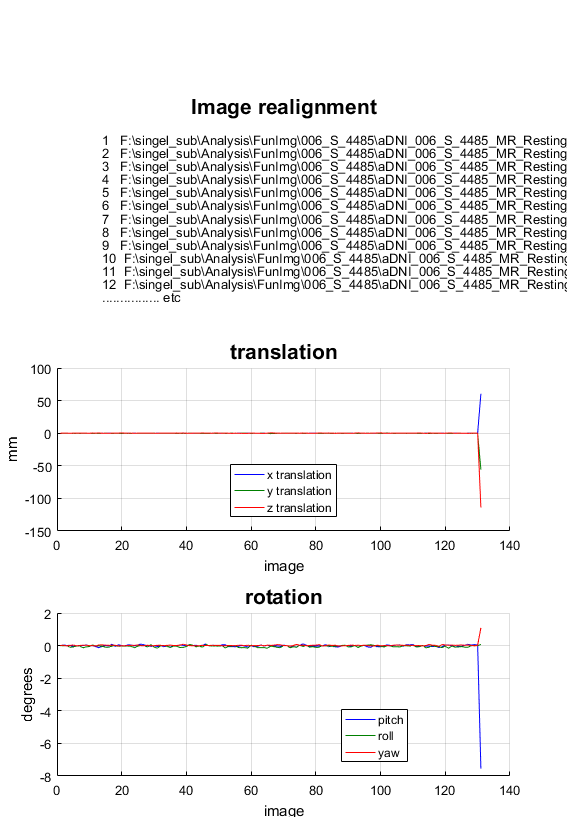


**2.8 Supplementary Fig 8**


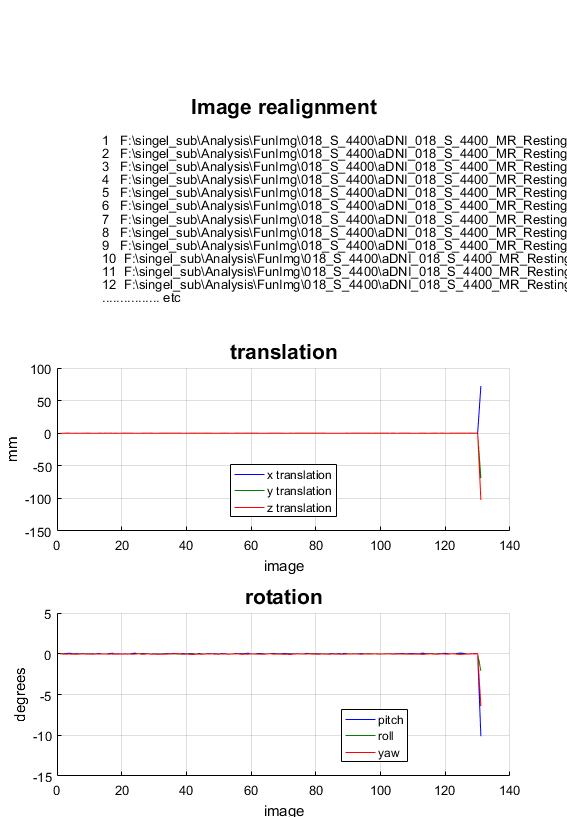


**2.9 Supplementary Fig 9**


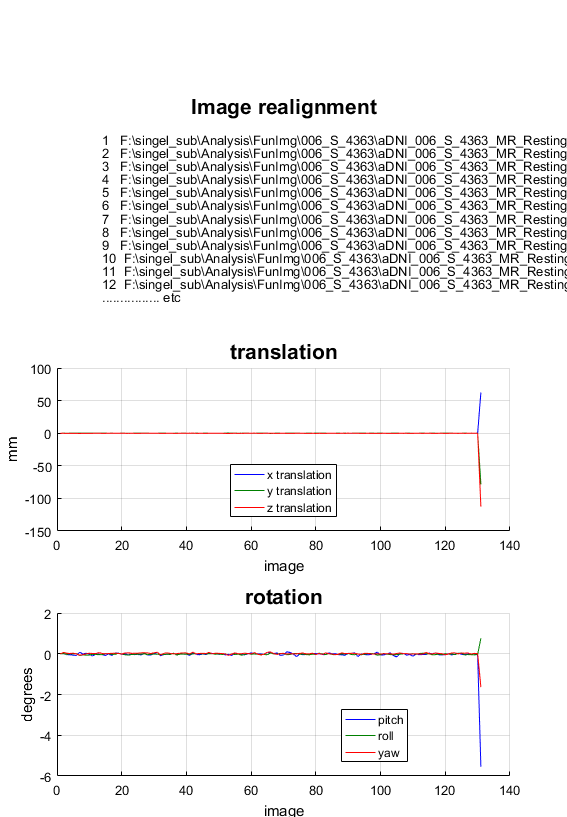


**2.10 Supplementary Fig 10**


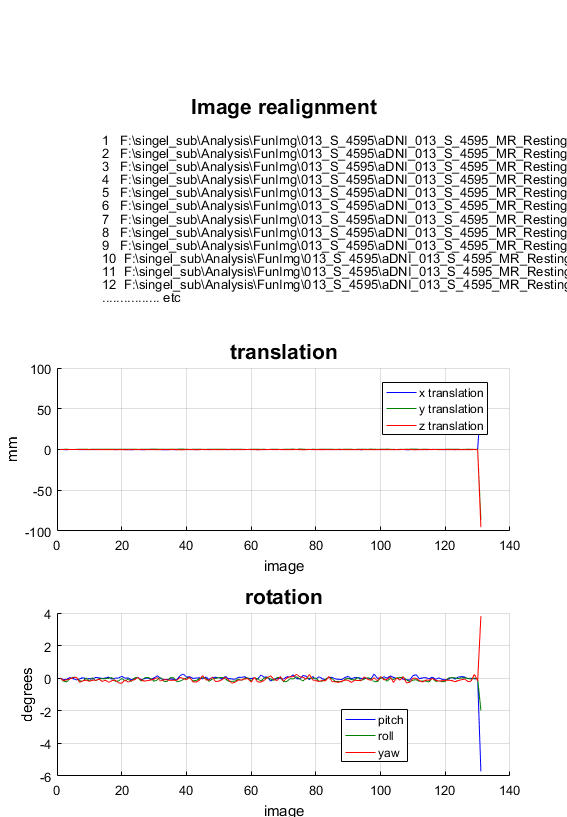


**2.11 Supplementary Fig 11**


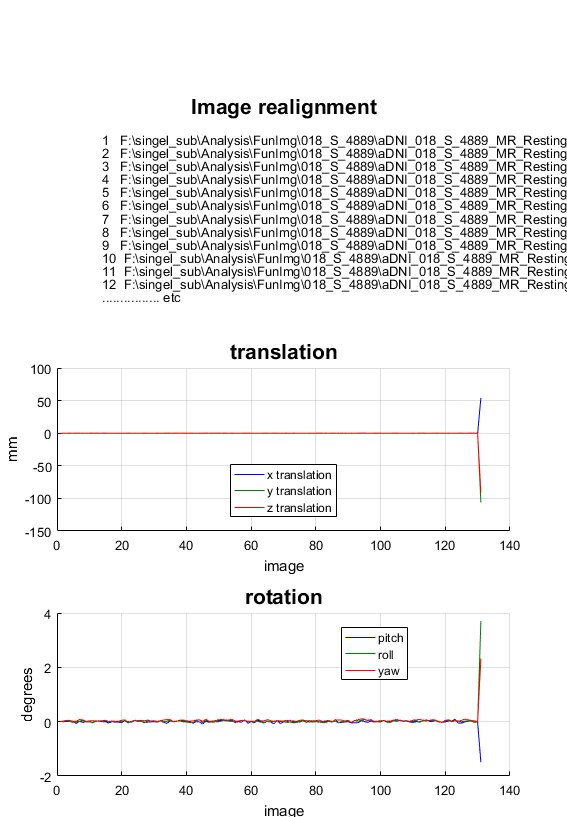


**References**

1. Benjamini Y, Hochberg YJJotRSS. Controlling the false discovery rate : a practical and powerful approach to multiple testing. 1995;57:289-300.

2. Firbank MJ, Coulthard A, Harrison RM, Williams EDJPiM, Biology. A comparison of two methods for measuring the signal to noise ratio on MR images. 1999;44:N261.

3. Lerski RA, Certaines JDDJMRI. Performance assessment and quality control in MRI by Eurospin test objects and protocols. 1993;11:817-833.

4. McRobbie, Radiology DWJBJo. The absolute signal-to-noise ratio in MRI acceptance testing. 1996;69:1045-1048.

5. Redpath, Radiology TWJBJo. Signal-to-noise ratio in MRI. 1998;71:704-707.

6. Sijbers J, Dekker AJD, Audekerke JV, Verhoye M, Dyck DVJMRI. Estimation of the noise in magnitude MR images. 1998;16:87-90.

7. Bodurka J, Ledden PJ, Gelderen PV, et al. Scalable multichannel MRI data acquisition system. 2010;51:165-171.

8. Dietrich O, Raya JG, Reeder SB, Reiser MF, Schoenberg SOJJoMRI. Measurement of signal-to-noise ratios in MR images: influence of multichannel coils, parallel imaging, and reconstruction filters. 2010;26:375-385.

9. Kaufman L, Kramer DM, Crooks LE, Ortendahl DAJR. Measuring signal-to-noise ratios in MR imaging. 1989;173:265-267.

10. Reeder SB, Wintersperger BJ, Dietrich O, et al. Practical approaches to the evaluation of signal-to-noise ratio performance with parallel imaging: Application with cardiac imaging and a 32-channel cardiac coil. 2010;54:748-754.

11. Zuo XN, Anderson JS, Bellec P, et al. An open science resource for establishing reliability and reproducibility in functional connectomics %J Data. 2013;1:140049.
